# A Global Health Survey of People Who Vape but Never Smoked: Protocol for the VERITAS (Vaping Effects: Real-World International Surveillance) Study

**DOI:** 10.2196/54236

**Published:** 2024-03-28

**Authors:** Jeffrey Zamora Goicoechea, Allison Boughner, Juan José Cirion Lee, Aman Mahajan, Kurt Yeo, Maris Sproga, Tasmin Patel, Claudio Saitta, Christopher Russell, Michael Coughlan, Pasquale Caponnetto, Riccardo Polosa

**Affiliations:** 1 International Network of Nicotine Consumer Organisations Vejle Denmark; 2 Asociación de Reducción de Daños del Tabaquismo Bogota Colombia; 3 Asociación de usuarios de vaporizadores y métodos de reducción de daños por tabaquismo de Costa Rica San José Costa Rica; 4 American Vapor Manufacturers Prescott, AZ United States; 5 South Carolina Vapor Association Charleston, SC United States; 6 México y el Mundo Vapeando Mexico City Mexico; 7 Vaping Saved My Life Benoni, Gauteng South Africa; 8 World Vapers Alliance Miami, FL United States; 9 Confidosoft Ltd, Leatherhead London United Kingdom; 10 ECLAT Srl University of Catania Catania Italy; 11 Russell Burnett Research and Consultancy Ltd Glasgow United Kingdom; 12 Section of Psychology Department of Science of Education University of Catania Catania Italy; 13 Center of Excellence for the Acceleration of Harm Reduction University of Catania Catana Italy; 14 Department of Clinical and Experimental Medicine University of Catania Catania Italy

**Keywords:** electronic cigarette, health effects, respiratory symptoms, survey, real-world use study, e-cigarette, e-cigarettes, smoke, smoker, smokers, smoking, vape, vaping, respiratory, pulmonary, cross-sectional, questionnaire, questionnaires, survey, surveys

## Abstract

**Background:**

There is only limited information about the health effects of regular vaping. Research on the health status of people who used to smoke faces the challenge that previous smoking may have caused unknown health effects. Only studies of people who vape but have never smoked combustible cigarettes can enable the detection of harms attributable to vaping. Large prospective studies of well-characterized electronic cigarette users with and without a history of combustible cigarette smoking are warranted to establish the long-term effects of regular vaping on respiratory health.

**Objective:**

We will conduct a global cross-sectional survey of individuals from 6 world regions. Respiratory symptoms will be assessed using a validated questionnaire—the Respiratory Symptom Experience Scale (RSES). Current vapers who are nonusers of other tobacco or nicotine products will be compared with matched controls who are nonusers of vapes and other tobacco or nicotine products.

**Methods:**

This will be a multicountry, cross-sectional internet-based survey of 750 adults aged ≥18 years who satisfy the criteria for inclusion in either a cohort of people who exclusively vape and who are nonusers of other tobacco or nicotine products (“vapers cohort”; target N=500) or a cohort of nonvapers who are also nonusers of other tobacco or nicotine products (“controls cohort”; target N=250). The primary end point of the study is the RSES score. RSES scores of people in the “vapers cohort” will be compared with those of people in the “controls cohort.” Additionally, the study will collect data to characterize patterns of vaping product use among the vapers cohort. Data collection will include information about the age initiation of using vape products, reasons for starting and continuing the use of vape products, specific types of products used, flavors and nicotine strengths of recently used products, as well as the frequency and intensity of product use in the past 30 days.

**Results:**

Participant recruitment started in April 2023, and enrollment was completed by November 2023 with 748 participants. Results will be reported in 2024.

**Conclusions:**

This will be the first study providing key insights into respiratory health effects associated with using electronic cigarettes in people who vape with no established use of combustible cigarettes or other tobacco or nicotine products.

**International Registered Report Identifier (IRRID):**

DERR1-10.2196/54236

## Introduction

Electronic cigarettes (ECs) are battery-powered electronic devices that vaporize a water-soluble nicotine solution for inhalation. They do not create smoke or use combustion to operate. These consumer products have been rapidly gaining ground on combustible cigarettes (CCs) among smokers because of their potential for harm reduction from cigarette smoke and smoking cessation [1-3], their competitive price [4,5], and because of allowing people who smoke to continue having a “smoking experience without smoking” [6-8].

Considerable controversy persists surrounding the use of these products, particularly concerning potential misuse by nonsmoking youths and their potential negative impact on respiratory health [9-11]. Many studies examining nicotine vaping face challenges in data analysis due to participants having a history of CC smoking. This history might contribute to some of the adverse effects reported in prior studies, as highlighted in recent papers [12-14].

Under normal operational conditions (without overheating or dry burning coils), ECs present a significant reduction in exposure to harmful constituents compared to CCs [15-17]. No discernible negative health effects solely attributed to EC use (commonly known as “vaping”) have been conclusively established [18-20]. Analytical chemistry and toxicology studies of vaporized chemicals suggest no imminent health risks, although concerns persist about potential long-term respiratory implications [21-23]. Hence, further investigation into the prolonged health impact of persistent vaping is warranted.

The respiratory system is the most likely site for any potentially harmful effects of constituents in EC aerosol emissions. Only a few clinical studies have investigated the impact of regular vaping. No deterioration in lung function, airway responses, and respiratory symptoms were observed in a 1-year prospective randomized controlled trial of “healthy” people who smoked and were invited to quit or reduce their cigarette consumption by switching to ECs [24,25]. Specifically, FEF25-75% (a sensitive measure of obstruction in the more peripheral airways) [24], nitric oxide (a noninvasive biomarker of airway inflammation in airway disease as well as in studies of environmental and occupational exposure) [25], and carbon monoxide (an exposure unique to smoking, among tobacco product uses, causing airway inflammation and cardiovascular disease) in the exhaled breath returned to normal limits [25]. Similar results were observed for those who continued to use ECs versus those who quit using them. In addition, restoration of lung defense has been shown in smokers who had switched to exclusive use of ECs; they exhibited mucociliary clearance efficiency similar to that of a never-smoker and a former smoker [26]. Overall, these preliminary studies do not appear to suggest negative respiratory health outcomes in people who smoke and have switched to ECs.

Researching individuals who switch from CC smoking to ECs and then quit presents challenges in understanding their health outcomes. Studying the health of former smokers is complicated due to unknown health effects caused by previous smoking. Precise information about their smoking history—duration, quantity, and puffing behavior—alongside a large sample size is necessary to accurately control for these effects. Recent work by Sargent et al [13] underscores the importance of appropriately accounting for smoking history. They used data from the PATH (Population Assessment of Tobacco and Health) study and, by adjusting for the cumulative amount of CCs smoked (pack-years), demonstrated how using more refined analytical approaches revealed a previously significant association as nonsignificant. This highlights the substantial residual confounding present when using basic binary measures instead of more comprehensive assessments.

Therefore, only studies of people who vape but have never smoked CCs or have only a limited smoking history (ie, those who have smoked fewer than 100 CCs in their lifetime) can enable the detection of harms attributable to EC use. Without studies of people who use ECs with no established smoking history, it will be impossible to distinguish the harms of vaping from neither vaping nor smoking. Concluding that vaping poses less risk than smoking is easy [11,15-20,27-29]. Quantifying the absolute health risks of vaping independent of smoking requires cleaner data.

In a small study of daily EC users who had never smoked, no noticeable changes in blood pressure, heart rate, lung function, respiratory symptoms, exhaled breath nitric oxide, exhaled carbon monoxide, and high-resolution computed tomography of the lungs from baseline were observed over an average 3.5-year observation period [30]. Daily exposure to EC aerosol emissions caused no significant changes in any of the health outcomes investigated, including measures of lung function and lung inflammation. Moreover, no significant structural abnormalities were identified in the high-resolution computed tomography of the lungs, and no respiratory symptoms were consistently reported. Some of the strengths of this study included prospective rather than retrospective data collection from study participants; the detailed vaping history and precise characterization of the study participants; as well as the use of a panel of different clinical, functional, and inflammatory measures during the study.

Large prospective studies of well-characterized EC users with and without a history of CC smoking are warranted to establish the long-term effects of regular vaping on respiratory health. We will conduct a global cross-sectional survey of individuals from 6 world regions. Respiratory symptoms reported by a cohort of individuals who vape with no established history of CC smoking or other tobacco or nicotine product use will be compared against those reported by a cohort of matched controls with no established history of vaping, smoking, or use of any other tobacco products.

The primary aim of this study is to test the hypothesis that chronic exposure to EC aerosols does not cause an increase in respiratory symptoms among people who vape with no established cigarette smoking or other tobacco and nicotine product use history. The secondary aims are to determine the feasibility of assembling a large cohort of individuals who vape with no other established tobacco or nicotine product use history to evaluate such effects for future studies and to characterize patterns of vaping product use behavior among this unique cohort.

## Methods

**Figure 1:**
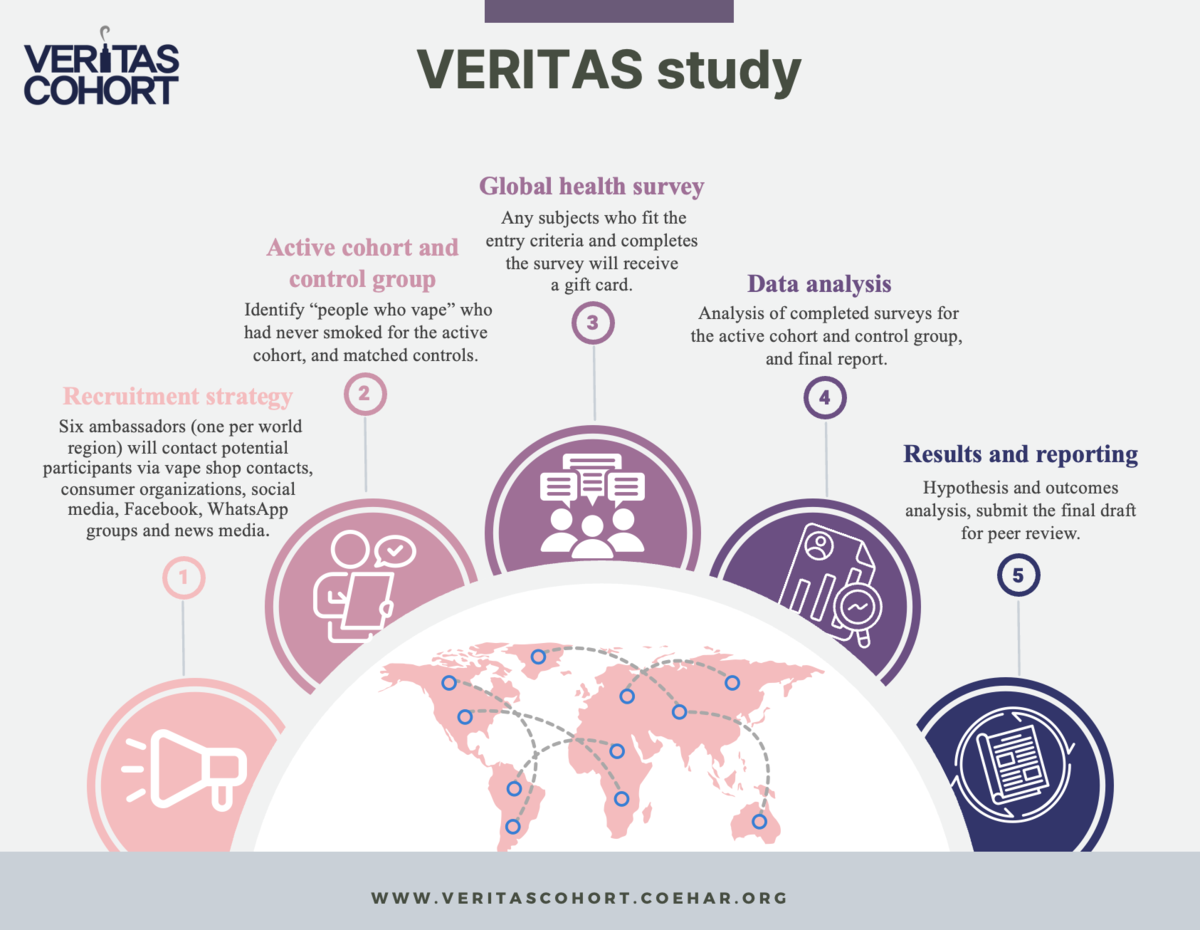
Study design. The infographic illustrates the different stages of the study.

An overview of the study is illustrated in Figure 1.

### Study Design

This will be a multicountry, cross-sectional, internet-based survey study. Individuals expressing an interest in participating in the study will be directed toward a website [31] with further information and will be screened using a brief questionnaire to assess their eligibility (Multimedia Appendix 1). All screening information gathered for contacts who are not potential recruits (ie, screen failures) will be discarded. Before conducting the survey, potential participants will be rescreened via telephone or email to confirm eligibility, to make sure they have an adequate understanding of the study, and to determine what language is required for the survey. Participants in the cohort will then be subjected to a web-based or mobile-based survey [32] using standard secure web-based survey tools, with central data collection. After completing the survey, participants will receive an email explaining how to redeem an Amazon or Take-A-Lot gift card code for an equivalent value of US $30, as appreciation for participating in the study.

### Ethics Approval

The study protocol has been reviewed and approved by the Ethic Review Board of Dipartimento di Scienze della Formazione Sezione di Psicologia at the University of Catania on November 25, 2020 (Ref 104/20).

### Population

This study plans to recruit approximately 750 individuals globally, who satisfy the criteria for inclusion in either a cohort of “people who vape and who are nonusers of other tobacco or nicotine products” (“vapers cohort”; target N=500) or a cohort of “nonvapers who are also nonusers of other tobacco or nicotine products (“controls cohort”; target N=250), as defined in the following sections.

### Recruitment

Carefully selected recruiters, the VERITAS (Vaping Effects: Real-World International Surveillance) ambassadors (AB, JJCL, AM, KY, and MS), will make sure to identify and screen individuals for the VERITAS Cohort Study. The VERITAS ambassadors will pursue recruitment through social media channels, WhatsApp groups, Google email groups, posters in vape shops and universities, recommendations from other participants, and recommendations from local vape shop owners in each of the 6 geopolitical world regions (ie, Africa and Middle East, North America, Latin and South America, Asia-Pacific, Western Europe, and Eastern Europe). Ambassadors will instruct vape shop owners to ask their regular clients about their smoking history and EC use patterns to identify potential participants. The reference or control group will comprise adults who never used any tobacco or nicotine products. Control group participants will be recruited primarily among vaping individuals’ circles of friends and acquaintances. They will be matched for age and sex to the extent possible. In the event of shortfalls from this recruiting pathway, the Veritas Ambassadors will recruit further controls.

VERITAS ambassadors will be acting as the main point of contact for study participants and will be coordinated by a project leader (JZG). A document detailing the ambassadors’ roles can be found on the VERITAS Cohort website [33].

### Eligibility Criteria

Eligibility to participate in this study will be assessed by a self-complete screener questionnaire administered immediately after participants provide informed consent to participate.

#### Inclusion Criteria

Eligibility for participation in the study is determined by the following criteria. All individuals who wish to participate must be aged ≥18 years and provide informed consent to participate in the study.

The vapers cohort should meet the following inclusion criteria:

1. Use at least 1 of 3 types of EC or vape—disposables, rechargeable with replaceable prefilled pods or cartridges, or rechargeable and refillable with e-liquid.
2. Have never smoked a CC *or* have smoked a CC *but* have not smoked more than 100 CCs in their lifetime [34] *and* have not smoked a CC in the past 3 months.
3. Has never used, *or* has never used more than once a week, any of 9 other tobacco or nicotine products listed below:
  Traditional cigars
  Cigarillos or filtered cigars
  Smoked tobacco in a hookah
  Smoked tobacco in a pipe
  Smokeless tobacco (including dip, spit, moist snuff, pouches, and chewing tobacco)
  Snus pouches
  Dissolvable tobacco
  Heated tobacco products (eg, iQOS, Glo, and Pulze)
  Tobacco-free nicotine pouches

The controls cohort should meet the following inclusion criteria:

1. Have never used, or have never used more than once a week, any of the 3 types of EC or vaping products listed above.
2. Same as the vapers cohort’s inclusion criterion 2.
3. Same as the vapers cohort’s inclusion criterion 3.

#### Exclusion Criteria

Individuals who do not satisfy the criteria for inclusion in either the vapers cohort or the controls cohort will be excluded.

### Study Procedures

All study procedures, from recruitment and obtaining consent to eligibility checks, questionnaires, data collection, and participant compensation, will be carried out exclusively online. The VERITAS web-based survey software (custom-based) will serve as the primary platform for data collection and displaying the Informed Consent Form. This software is intended not only to optimize the presentation of materials for study participants but also to facilitate data submission across various devices, such as laptops, smartphones, and tablets, while being compatible with all web browsers, including Chrome, Safari, Firefox, and Internet Explorer. By adopting this approach, study participants will have the freedom to engage in the research at their convenience, within the privacy of their choosing, and using their preferred device. As a result, participants will be empowered to take part in the study on their terms, in a comfortable setting, and at a time that suits them best.

### Web-Based Survey Questionnaires

After prescreening by the ambassadors, eligible individuals who consent to participate will be directed to the study website [32], where they will be subject to a 2-step verification process. When participants reach the survey platform, they will click “Don’t have an account? Register”; participants will register their email addresses. Following registration, participants will receive a “Welcome email” and a link to re-enter the survey platform, where they will be asked for their phone number to receive a confirmation code via SMS. After confirming their email addresses, they will start filling out the survey, which will consist of sociodemographic data, medical history, as well as tobacco and nicotine product use behavior. Details about the VERITAS survey document can be shared upon specific request to the corresponding author. The information collected via the web-based questionnaire will help to characterize or describe the unique cohort. Individuals will then be included in the vapers cohort or the controls cohort or they will be excluded based on their responses to questions about their past and current use of cigarettes, EC or vapes, and other tobacco and nicotine-containing products.

Individuals in the vapers cohort will then be asked questions about their historical and current patterns of use of 3 types of vaping products (ie, disposable vaping products, rechargeable prefilled pod vaping products, and rechargeable refillable vaping products). For each vaping product category, data will be collected on 12 outcomes, as applicable: (1) age of first use; (2) age of initiation, when started to vape more than once a week; (3) the number of product units used in their lifetime; (4) the number of product use days in the past 30 days; (5) length of time (eg, years or months) of vaping more than once a week; (6) nicotine content of products used; (7) flavor categories vaped more than once a week; (8) number of flavors vaped more than once a week; (9) name of favorite flavor used in the past 30 days; (10) number of product units used in the past 30 days; (11) reasons for initiating product use (free-text response); and (12) reasons for current product use (free-text response). The VERITAS survey document can be shared upon specific request to the corresponding author.

All participants will then complete the Respiratory Symptom Experience Scale (RSES), a validated scale that asks participants to rate the frequency with which they experienced 5 respiratory symptoms in the past 30 days [35]. The 5 symptoms rated are as follows: (1) morning cough with phlegm or mucous, (2) coughing frequently throughout the day, (3) shortness of breath that makes it difficult to do normal daily activities, (4) becoming easily winded during normal daily activities, and (5) wheezing or whistling in the chest at times when not exercising or doing other physically strenuous daily activities. Rating for each symptom is made on a 5-point scale, where 1=Never (0 out of the last 30 days); 2=Rarely (1-5 days); 3=Occasionally (6-15 days); 4=Most days (16-29 days); 5=Every day (all 30 out of the last 30 days). A mean RSES score will be calculated by averaging the 5-item scores. The RSES can be found in the Multimedia Appendix 1.

Lastly, questions will assess participants’ sex, country of residence, employment status, highest educational attainment, height, and weight.

If necessary, surveys will be translated into the local language by the ambassador for countries where not all participants are fluent in English. Data will be collected on whether the participant completed the survey in English, Spanish (translation available on the platform), or their local language (in case it is determined that some questions of interest had some important difference in nuance when translated).

### Study End Points

The primary end point of the study is the RSES score [35]. The objective of the study is to compare the RSES scores between people who vape with no established CC smoking history (ie, the “vapers cohort”) and people who have never vaped nor smoked (ie, the “controls cohort”).

Additionally, the study will collect data to carefully characterize people who vape with no established CC smoking history in the “vapers cohort.” Data collection will include information about the age of initiation of using vaping products, reasons for starting and continuing use of vaping products, specific types of products used, flavors and nicotine strengths of recently used vaping products, as well as the frequency and intensity of product use in the past 30 days.

### Statistical Analyses and Reporting

The primary objective of the study is to retrospectively estimate the effect of current, regular use of vaping products on respiratory symptom experience in the past 30 days, measured in this study by the mean score (0 to 5) on the 5-item RSES [35]. Details of sample size calculation and power analysis to detect differences in respiratory symptoms (ie, the RSES scores) between the “vapers cohort” and the “controls cohort” are included in Multimedia Appendix 1. In brief, with 75 participants per group, a study should be adequately powered to detect a difference of 0.57, the proposed minimal important difference, with a power of 98%. However, to detect a more conservative difference of 0.40, a study with 75 individuals per group would still have a power of 89%. The target study population will consist of approximately 500 exposed individuals and 250 matched controls–approximately 83 people who vape per each of the 6 world regions. If the initial recruitment falls short of the goal, or in the case of substantial early loss to follow-up, rolling enrollment will be considered to make up the deficit.

Data analysis of the RSES scores will be both descriptive and inferential in nature. The RSES scores and the demographic characteristics of the vapers cohort and the controls cohort will be descriptively summarized. We will test the null hypothesis that the RSES scores will not significantly differ between the vapers cohort and the controls cohort when adjusted for demographic differences. This hypothesis will be tested through a one-way between-subjects Analysis of Covariance in which the mean RSES score will be entered as the dependent variable, “Cohort” will be entered as the between-subjects independent variable, and 4 demographic variables—age, sex, education, and employment status—will be entered as covariates. Inferential and descriptive statistics will be reported for the Cohort variable adjusted for the effects of the covariates. *P* values <.05 will be considered statistically significant.

The secondary objective of this study is to characterize the use of different vaping products by the vapers cohort. Data analysis of this objective will be purely descriptive in nature. Descriptive summary statistics will be reported for all questionnaire measures of vapers’ use of vaping products within each of the 3 product categories—disposable vapes, rechargeable prefilled pod vapes, and rechargeable refillable vapes. Data on the following variables will be descriptively summarized for the subsets of participants within the vapers cohort who are current users of each product category, as follows: number of current users, age of first use, age of first regular use, number of products used in a lifetime, number of product use days in the past 30 days, time of regular product use, the nicotine concentration of products used typically, type of e-liquid flavors used in the past 30 days, number of e-liquid flavors used regularly in the past 30 days, and number of products used in the past 30 days.

Descriptive summary statistics for continuous variables will include the number of participants in the population of interest (N), the number of participants in the population of interest with nonmissing data on the variable or measure (n), as well as appropriate measures of central tendency (eg, mean and median) and dispersion (eg, SD, SE, range, and IQR) for the observed data. Descriptive statistics reported for categorical variables or measures will include the total number of participants in each subject group who provided nonmissing responses, the proportion of each subject group who selected each categorical response option, and when appropriate, 95% CIs around the proportion of participants who selected each categorical response option.

Additional data analyses may be conducted where data on variables or measures are filtered or stratified by other independent variables of interest. If undertaken, the reporting of descriptive summary statistics for each variable or measure filtered and stratified by additional independent variables of interest will closely resemble the reporting of the planned analyses described above. All data analyses will be conducted using the IBM SPSS (version 27 or higher; IBM Corp) statistical software. Data tables not presented in primary reports or in the manuscript will be presented in supplementary files.

## Results

Participant recruitment started in April 2023, and enrollment was completed by November 2023 with 748 participants. Results will be reported in 2024.

## Discussion

Research into the respiratory health effects of regular vaping is limited [11,36,37]. Previous research investigating the health status of individuals who used to smoke faces challenges due to unknown health effects caused by prior smoking. Consequently, studies involving individuals who solely vape with no established smoking history are essential to identify potential harms attributed to vaping. A small prospective study of daily EC users who never smoked CCs demonstrated no significant alterations in lung function, inflammation, or structural abnormalities observed in lung scans [30]. Moreover, consistent respiratory symptoms were not reported in this study [30]. However, large longitudinal studies focusing on well-characterized EC users, with and without a history of CC smoking, are necessary to establish the long-term effects of regular vaping on respiratory health.

This proposed study aims to be the first investigation exploring the association between respiratory health effects and the use of ECs in a large group of individuals who exclusively vape, with no established CC smoking history. Additionally, the research will delve into potential confounding factors that could influence any association identified between vaping and respiratory symptoms.

Several noteworthy features distinguish this study. First, by enlisting participants globally, it intends to capture diverse experiences and behaviors related to EC use across different countries and regions. Second, the study’s focus on respiratory symptoms and in-depth characterization of vaping product use in a real-world context enhances its generalizability. Third, the study sample has been adequately powered to detect minimum clinically important differences in respiratory symptoms between exclusive vapers and those who neither vape nor smoke. Finally, the study implements a stringent 2-step verification process to deter potential abuse by unauthorized users attempting to exploit the possibility of receiving a gift card and uses manual analysis to identify and verify legitimate participants.

Although this study promises important insights into respiratory symptoms and vaping behaviors among a culturally diverse group of adults who exclusively vape, it should be considered within the context of several limitations. First, its cross-sectional design limits the ability to draw conclusions about the causal relationship between vaping and respiratory symptoms. Longitudinal studies will be crucial to discern the prospective effects of vaping behavior on respiratory health. Second, the study’s reliance on nonprobability sampling methods may introduce a self-selection bias, impacting the generalizability of the findings to the broader population of exclusive vapers. However, given the rarity of this population subgroup, these methods were deemed the most practical approach to gathering a sufficient sample. Third, relying on a single respiratory health assessment (ie, RSES) based on self-report might introduce biases and inaccuracies. Participants might hesitate to disclose certain information or be influenced by social desirability bias, potentially affecting the data quality. Lastly, variations in survey interpretation due to linguistic, cultural, or regulatory differences across countries could lead to diverse responses, impacting the consistency and accuracy of the data collected for the research question.

The results obtained from the VERITAS study may offer crucial insights into the impact of vaping on health outcomes, providing a foundation for implementing essential public health measures. These findings can be effectively used within public health sectors and policies to address health concerns related to vaping, establishing necessary preventive measures to safeguard vulnerable populations. Moreover, the study methodology stands as a valuable reference for forthcoming research. Building such unique cohorts could serve as a pivotal resource for future studies, enabling the thorough examination of safety concerns and the quantification of potential risks or harm associated with vaping.

## Appendix

Multimedia Appendix 1 Cohort study questionnaire.

## Abbreviations

CC: combustible cigarette
EC: electronic cigarette
PATH: Population Assessment of Tobacco and Health
RSES: Respiratory Symptom Experience Scale
VERITAS: Vaping Effects: Real-World International Surveillance
