# Supplementary material for: A Global Health Survey of People Who Vape but Never Smoked: Protocol for the VERITAS (Vaping Effects: Real-World International Surveillance) Study
Source: JMIR Res Protoc. 2024 Mar 28;13:e54236. doi: 10.2196/54236 (PMC11009848; doi:10.2196/54236)
Supplement: Multimedia Appendix 1 [file resprot_v13i1e54236_app1.docx]

**APPENDIX**

**TABLE OF CONTENTS**

TERMS OF USE...................................................................................................................3

1

ELIGIBILITY...............................**ERRORE. IL SEGNALIBRO NON È DEFINITO.**

1. 1.1  Demographics ............................................... **Errore. Il segnalibro non è definito.**
2. 1.2  Cigarettes ...................................................... **Errore. Il segnalibro non è definito.**
3. 1.3  Electronic Nicotine Vaping Products............. **Errore. Il segnalibro non è definito.**
4. 1.4  Other Tobacco and Nicotine Products ........... **Errore. Il segnalibro non è definito.**

DISPOSABLE VAPING PRODUCTS**ERRORE. IL SEGNALIBRO NON È DEFINITO.** RECHARGEABLE PRE-FILLED POD VAPING PRODUCTS............... **ERRORE. IL**

2 3

**SEGNALIBRO NON È DEFINITO.**

4 RECHARGEABLE REFILLABLE VAPING PRODUCTS**ERRORE. IL SEGNALIBRO NON È DEFINITO.**

1. 5  RESPIRATORY SYMPTOMS.....**ERRORE. IL SEGNALIBRO NON È DEFINITO.**
2. 6  DEMOGRAPHICS .......................**ERRORE. IL SEGNALIBRO NON È DEFINITO.**
3. 7  TERMINATION MESSAGE........**ERRORE. IL SEGNALIBRO NON È DEFINITO.**
4. 8  END MESSAGE ..........................**ERRORE. IL SEGNALIBRO NON È DEFINITO.**

This document contains confidential and proprietary information of Russell Burnett Research & Consultancy Ltd (RBRC) and ECLAT Srl.

**DISCLAIMER AND TERMS OF USE**

This document is the intellectual property of Russell Burnett Research and Consultancy Ltd (RBRC) and ECLAT Srl. This document contains confidential and proprietary information of RBRC and ECLAT Srl. Therefore, viewing and use of the questionnaire must be authorized by RBRC or ECLAT Srl.

**All requests to share this document should be directed by email to Sebastiano Antonio Pacino, President of ECLAT Srl. at: info@eclatrbc.it**

**VERITAS Cohort Study**

**Pre-Screening Questionnaire**

**This brief questionnaire serves to quickly identify potential participants for VERITAS Cohort study. The recruiter must use it when he/she approaches potential/prospective participants. This can contribute to recruitment success.**

**Questions:**

1. Are you 18 years old or older?
2. Are you currently using electronic cigarettes?
3. Have you been vaping daily for at least three months?
4. Did you smoke any form of combustible tobacco before starting to vape? If so, have you smoked less than 100 cigarettes in your lifetime?
5. Would you be interested in participating in a study? It is a multi-country internet-based survey of adults.
6. If you are interested in participating in the survey, will you accept answering questions regarding age initiation of using vape products; reasons to start using and continuing using vape products; specific types of products used; flavour preferences; nicotine levels; frequency and pattern of use; and self-perceived respiratory health?
7. Do you have an email and phone number to confirm and validate your registration on our platform?

**The Respiratory Symptom Experience Scale (RSES): Questionnaire to assess respiratory symptoms**

In the last 30 days, how many times have you experienced the following?

|  | Never  (0 days of last 30 days) | Rarely  (1-5 days) | Occasionally  (6-15 days) | Almost every day  (16-29 days) | Every day  (all 30 days of the last 30 days) |
| --- | --- | --- | --- | --- | --- |
| Cough in the morning with phlegm or mucus |  |  |  |  |  |
| Cough frequently during the day |  |  |  |  |  |
| My shortness of breath makes it difficult to do normal daily activities, such as climbing a flight of stairs or carrying a heavy object |  |  |  |  |  |
| I am easily out of breath during normal daily activities (for example, doing laundry or carrying groceries) |  |  |  |  |  |
| Sometimes, I hear a hiss or whistle in my chest when I’m not doing exercise or other physically strenuous daily activities (for example, at rest) |  |  |  |  |  |

**Administration and Scoring**

Each item of the RSES is administered reminding participants of the recall period: "In the last 30 days, how many times have you experienced the following?" The RSES items are administered in a fixed order and the order of the answer options is also fixed. It is recommended that researchers use the raw scores of RSES items to calculate a composite. A composite score is calculated by taking the average of the 5 items of the RSES. If an item is missing, no composite should be calculated.

**Power analysis to detect differences in respiratory symptoms between two groups**

The following graphs show the power to detect a range of group differences (0.30 to 0.80 in absolute differences), for two sample sizes: 60 per group or 75 per group. The graphs also use three different estimates of the pooled standard deviation.

- The first set is based on the pooled SD from the JCross study comparing Switchers and Smokers (Pooled SD =0.59). This represents the standard way of approaching power analysis.
- The second is more conservative, using the larger of the two SDs from the Smoker group in JCross as an estimate of the pooled SD (SD=0.76).
- The third uses the pooled SD estimate from the RSES validation study, which is larger than either of the other two estimates (pooled SD=0.87). This is likely much inflated by the fact that the study deliberately over-represented individuals with respiratory disease, including COPD. As a result, this is likely to underestimate power for a study that does not do such over-representation.

NB Note: the graphs differ in the range of the Y-axis (an unalterable feature of the power analysis software), so should not be visually compared, but read out quantitatively against the Y axis.

Bottom line: even with an N of 60 per group, a study should be adequately powered to detect a difference of 0.57, the proposed MID (Power is 95% to 99%, based on the two SD estimates from JCross). However, the RSES study only established that the MID is no higher than 0.57; it may be lower. It would therefore be prudent to power a study to detect differences smaller than 0.57. To detect a difference of 0.40, a study with 60 per group would still have power of 82% to 96%, depending on the estimate of the SD, but upping the sample size to 75 per group would increase power into the 89% to 98% range. More is better, as always.

N=60, Pooled SD = 0.59 (from JCross)


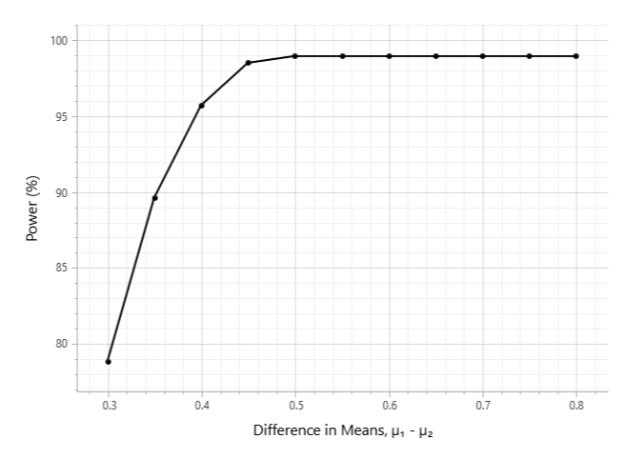


N=75, Pooled SD = 0.59 (from JCross)


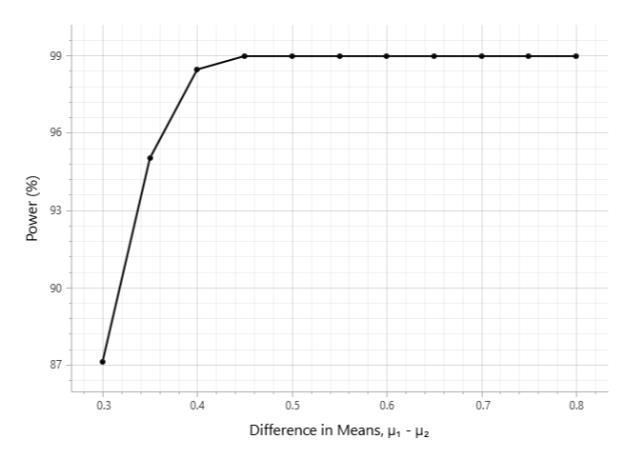


N=60, larger SD = 0.76 (from JCross)


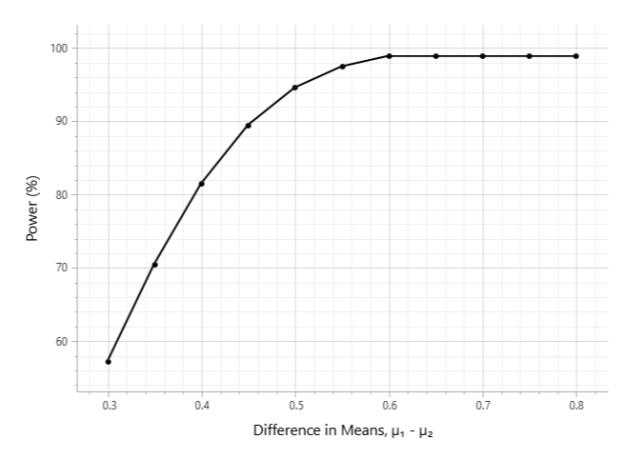


N=75, larger SD = 0.76 (from JCross)
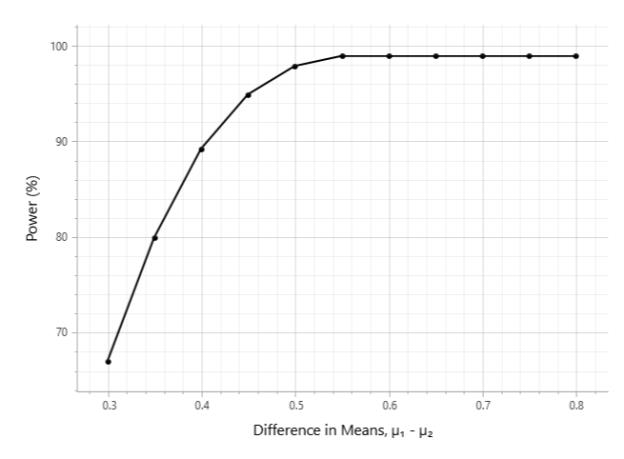


N=60, Pooled SD = 0.87 (from RSQ study)


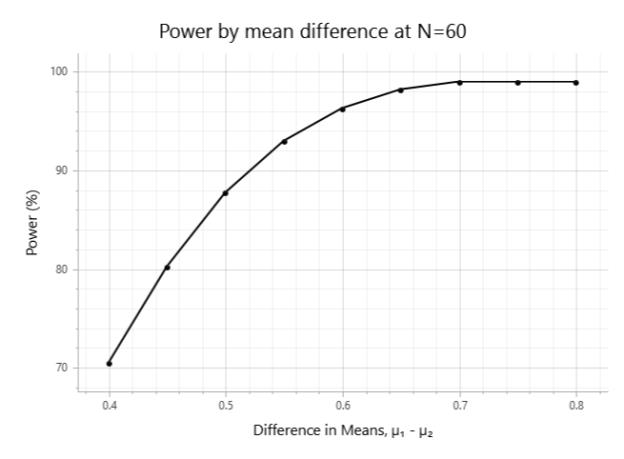


N=75, Pooled SD = 0.87 (from RSQ study)


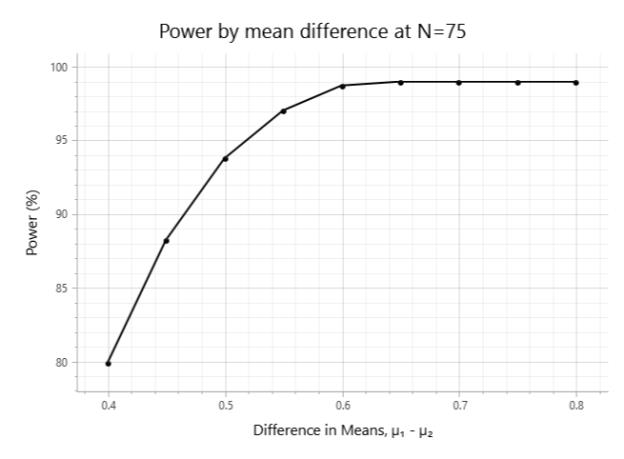


# Institutional website environment

**General remarks**

The institutional website for the Veritas Cohort study (<https://veritascohort.coehar.org>)  is a valuable resource for study participants, researchers, and the general public. It provides information about the study, its goals and the team (Project leader and local ambassadors). It also includes a FAQ section that answers common questions about the study. It also serves as an authoritative resource for ambassadors in the recruitment process.

The website will be created through an extensive copywriting process involving multiple stakeholders. This process ensured that the website is clear, concise, and accurate.

The website will be designed to be easy to use and navigate and it will be regularly updated with the outcomes from the study.

**Veritas Cohort website is powered by Wordpress.**

WordPress is the most popular CMS (Content Management System) in the world, powering over 40% of all websites online. It is a powerful and flexible platform that can be used to create any type of website, from a simple blog to a complex e-commerce store.

The technologies we used to host the website include:

**Nginx**: Nginx is an open-source **web server** that is a popular alternative to Apache. It is more lightweight and more efficient than Apache, which makes it a good choice for websites with high traffic. In this case we used it as a reverse proxy for Apache. This means that Nginx can be used to route traffic from visitors to Apache. This can be useful for a number of reasons, including:

*Performance*: Nginx can be used to cache static content, which can improve the performance of the website.

*Security*: Nginx can be used to filter traffic and block malicious requests, which can improve the security of the website.

*Scalability*: This is not our use case because we are not using containerization and or multiple instances, but Nginx can be also used to scale the website to handle more traffic.

**PHP 8.1** : PHP is a **server-side scripting language** that is used to create the website. It is used to generate the HTML that is displayed to visitors.

**MariaDB 10.11**: MariaDB is an open-source relational **database** that is a fork of MySQL. It is a good choice for websites with high traffic and has several features that make it faster and more secure than MySQL.

The website is hosted on a **dedicated server** that is entirely managed by us using the **AWS** IaaS service (**EC2 t2.xlarge Instance**). This means that we have full control over the server, including hardware, software, and operating system. We can install any software we want and customize it to our needs. This allows us to have a level of control and flexibility that would not be possible with a typical cheap shared hosting service.

# **Engineering environment**

#### Web

##### Flutter 3.7

Flutter is Google's UI toolkit for building beautiful, natively compiled applications for iOS, Android, web, desktop, and embedded devices from a single codebase.

Fast Development - Paint your app to life in milliseconds with Stateful Hot Reload. Use a rich set of fully customizable widgets to build native interfaces in minutes.

Expressive and Flexible UI - Quickly ship features with a focus on native end-user experiences. The layered architecture allows for full customization, which results in incredibly fast rendering and expressive and flexible designs.

Native Performance - Flutter’s widgets incorporate all critical platform differences such as scrolling, navigation, icons, and fonts, and your Flutter code is compiled to native ARM machine code using Dart's native compilers.

We used Flutter for the front end of the participants' survey questionnaire forms.

##### VS Code

Visual Studio Code is a freeware source-code editor made by Microsoft for Windows, Linux, and macOS. Features include support for debugging, syntax highlighting, intelligent code completion, snippets, code refactoring, and embedded Git. Users can change the theme, keyboard shortcuts, and preferences, and install extensions that add additional functionality.

VS Code is used for the coding for the website.

##### Angular 10.2.5

Angular is an open-source front-end framework developed by Google for creating dynamic, modern web apps. First introduced in 2009, the framework has gained huge traction to eliminate unnecessary code and ensure lighter & faster apps. Angular helps build interactive and dynamic single-page applications (SPAs) with its compelling features including templating, two-way binding, modularization, RESTful API handling, dependency injection, and AJAX handling. Designers can use HTML as a template language and even extend HTML syntax to easily convey the components of the application.

One of the biggest advantages of Angular is that it is supported by Google. The best part about it is Google’s Long-Term Support (LTS). This sheds light on Google’s plan to stick with it and further scale up the Angular ecosystem.

It is used for the front end of the admin site.

##### Dot NET Core 3.1

Dot NET Core is a robust and powerful development framework maintained by Microsoft. It can be used to develop applications on any platform. Often it is used for cloud applications or refactoring large enterprise applications into microservices.

The main advantages of the .NET core ecosystem are :

- Dot NET Core is open source.
- Dot NET Core feasibly supports Cloud and IoT applications.
- Dot NET Core can be used on a variety of platforms (macOS, Linux, Windows).
- Dot NET Core does not require expensive Windows/Server licenses and Windows systems for use.
- Increased security with Dot NET Core.
- Dot NET Core enables top app performance.

It is used to create the API for both admin and participant sites.

##### SQL 2012

SQL Server is a relational database management system (RDBMS) developed by Microsoft. Being a database server, its primary function is to store and retrieve the data as and when requested by other software applications. Administering the Microsoft SQL server database can help you optimize as well as maintain your server’s performance while ensuring its recoverability and availability. The main advantages of SQL servers are installation is streamlined, security features are better, enhanced performance, and lower cost of ownership.

It is used for the database, where all the data of users and surveys are stored.

#### Tools/Service

##### Firebase Authentication

Firebase Authentication is a service provided by Google's Firebase platform that allows users to easily add user authentication and identity management to applications. It offers a secure and reliable way to authenticate users and manage their login credentials.

Firebase authentication is used to authenticate the users to log in and fill up the survey.

##### GitHub

GitHub, Inc. is a provider of Internet hosting for software development and version control using Git. It offers the distributed version control and source code management (SCM) functionality of Git, plus its features. It provides access control and several collaboration features such as bug tracking, feature requests, task management, continuous integration, and wikis for every project.

GitHub is used for managing and versioning the code throughout the development process.
